# Liverpool and Venereal Disease

**Published:** 1921-01-01

**Authors:** 


### Liverpool and Venereal Disease.

An interesting and notable feature of the new Corporation Bill of the Liverpool City Council is that part of it which seeks to secure that persons suffering from venereal disease shall receive medical treatment. The Bill (*a*) makes it obligatory on such person, who is or has reason to believe that he is suffering from such disease, to consult a medical adviser, and carry out the treatment prescribed by him; (*b*) enables the sufferer to change his medical adviser and to continue treatment with another medical adviser; (*c*) provides for a certificate being issued in due course by his medical adviser to the patient that he had ceased to be liable to convey infection; (*d*) in the event of the sufferer not continuing the treatment until he is so certified, makes provision that the medical officer of health shall be informed of the fact by the medical adviser; (*e*) provides that any parent, guardian or person in charge of any child or mentally defective person suffering from venereal disease, who knows, or has reason to believe that they are so suffering, shall cause them to be treated by a medical adviser.

It is made a penal offence for any person knowingly to infect any other person with venereal disease, or knowingly to do or permit any act likely to lead to such infection of any other person. Provision is also made for the removal to a hospital or building provided by the Corporation of persons suffering from venereal disease on discharge from any hospital, workhouse, gaol, asylum, or other public institution until he has ceased to be liable to convey infection. The measures proposed by Liverpool appear to constitute a bold and direct challenge to the prevalence of this serious and widespread evil.

